# An update on the variations of the orbital blood supply and hemodynamic

**DOI:** 10.1007/s00276-016-1776-9

**Published:** 2016-11-09

**Authors:** Eugenio Bertelli, Marì Regoli, Sandra Bracco

**Affiliations:** 1 Department of Molecular and Developmental Medicine, University of Siena, Via Aldo Moro 2, 53100 Siena, Italy; 2 Unit of Neuroimaging and Neurointervention (NINT), Department of Neurological and Sensorineural Sciences, Azienda Ospedaliera Universitaria Senese, Policlinico “Santa Maria alle Scotte”, Siena, Italy

**Keywords:** Orbit, Ophthalmic artery, Anastomosis, Hemodynamic, Visibility index

## Abstract

**Purpose:**

Several variations of the arterial blood supply of the orbit have been reported over the years. This review is aimed to provide an update focusing on three important issues: (a) variations of the ophthalmic artery origin; (b) contribution of the external carotid artery to the orbital blood supply; (c) orbital hemodynamic.

**Methods:**

A PubMed and Google search was carried out with the following keywords: ophthalmic artery origin, ophthalmic artery anastomoses and ophthalmic artery anatomy.

**Results:**

The site of origin of the ophthalmic artery displays a limited number of variations. However they are important as they are also associated with course variations. Anastomoses between the ophthalmic artery and the external carotid artery are numerous and many of them can acquire clinical relevance. Records on their anatomic frequency are limited. Orbital hemodynamic variations are a poorly studied subject. Recent investigations in children have unveiled unexpected variability and instability in the way the blood flows through the orbit.

**Conclusions:**

The orbit shows several possible arterial variations. Some of them have a profound influence on its hemodynamic at least in children. More studies are required to ascertain if the hemodynamic variability observed in children can be pinpointed also in adults.

## Introduction

The anatomic variations of the arterial blood supply can be challenging in several clinical settings and particularly for orbital surgeons, neurovascular interventionalists and neuroradiologists. New investigations have recently added a body of valuable information that we believe it has come the time to sum up. Novel data on the anastomoses occurring between the external carotid artery (ECA) and the internal carotid artery (ICA) via the ophthalmic artery (OA) have been produced. In addition to their angiographic demonstration, a survey on the frequency of visualization has been produced for the first time [10]. Knowledge of the incidence that characterizes a certain vascular pattern provides clinicians and neurovascular interventionalists with a useful reference when searching for connections between the internal and external carotid systems. At first sight, angiography looks the perfect tool for this purpose as it is usually considered the gold standard to visualize blood vessels. However, when it comes to anastomoses, it seems that its efficacy is suboptimal. Apparently, some of them may appear (i.e. become detectable) under particular hemodynamic circumstances [5, 16]. Thus, it is important to ascertain how powerful angiography is to unveil orbital anastomoses. A way to achieve this task is to compare angiographic studies, which provide the frequency of visualization, with dissection-based investigations, which provide information on the true anatomic frequency of the anastomoses. By the combination of these data a numerical index can be generated which objectively measures the angiographic power to demonstrate each anastomosis. This parameter, referred to as the visibility index (VI) (see materials and methods), has been recently introduced to ascertain the ability of angiography to visualize and identify intraorbital arteries [9]. Unfortunately, as it will become apparent in the next paragraphs, in spite of the fact that orbital anastomoses are acknowledged of pivotal importance in several clinical scenarios [5, 17, 34], only limited information on their frequency are available, so that the VI can be calculated just in a small number of cases.

Still on the subject of the orbital blood supply, a novel field of interest that is opening up is the short-term hemodynamic variations that may occur. This mostly unexplored issue is the result of the many anastomoses occurring between the OA and the ECA that provide pathways for the internal and external carotid systems to compete for the orbital blood supply [10]. A glimpse into the matter was given in orbits of children affected by intraocular retinoblastoma [5]. In these patients, the repeated sessions of intra-arterial chemotherapy offered the chance to explore the short-term hemodynamic changes unlikely occurring secondary to the pathology still restricted inside the eyeball [5]. Though *tout*-*court* exportation of observations carried out on children to adults would be certainly incorrect, this study should be considered an interesting starting point to induce investigators to pursue the matter. To sum up, this review is focused on the following points:

1. Variations of the OA origin,
2. Contribution of the ECA to the orbital blood supply via anastomoses with the OA,
3. Orbital hemodynamic balance between ECA and ICA.

## Materials and methods

A PubMed and Google search was carried out with the following keywords: OA anatomy, OA origin, OA anastomoses. The reference lists of the relevant articles were carefully checked to extend the results of the electronic search.

To measure the power of angiography to visualize and identify vessels/anastomoses, a novel numerical index, the VI, is employed [9]. The VI is the ratio between the frequency of the angiographic visualization of a vascular structure (vessel or anastomosis) and its true anatomic incidence, the latter one picked up from the most reliable (in terms of number of anatomic samples employed) previously published dissection-based studies. Only when the frequency of angiographic visualization matches exactly the true anatomical incidence, the VI is equal to 1. VI values lower than 1 indicate that the identification by angiography of a given vessel/anastomosis can be missed in a variable number of cases in spite of its presence. Therefore, the VI of a given vessel/anastomosis can be calculated only when the frequency of detection by angiography and the anatomic incidence are both known.

## Development of the orbital blood supply

**Fig. 1:**
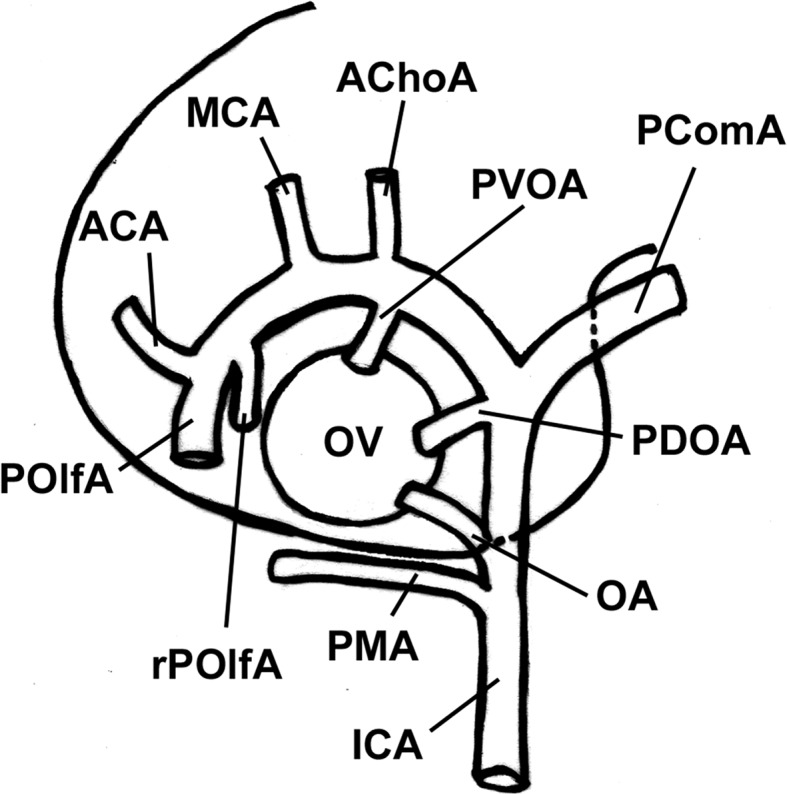
Schematic drawing of the arteries of the forebrain with special reference to the branches serving the optic vesicle in the embryo. The StA is not shown as it does not contribute to the blood supply of the optic vesicle. *PMA* primitive maxillary artery, *rPOlfA* recurrent primitive olfactory artery, *PVOD* primitive ventral ophthalmic artery, *PDOA* primitive dorsal ophthalmic artery, *OA* ophthalmic artery, *ACA* anterior cerebral artery, *POlfA* primitive olfactory artery, *ICA* internal carotid artery, *MCA* middle cerebral artery, *OV* optic vesicle, *AChoA* anterior choroidal artery, *PComA* posterior communicating artery (i.e., caudal division of the primitive ICA) Modified from Padget [51]

A brief account of Padget’s seminal work on the development of the cranial arteries [51] is worth to better understand the variations of the orbital blood supply. This is even more important, since the OA development has been frequently a matter of dispute [33] and it has recently been re-evaluated in light of a more attentive reading of Padget’s work [16, 18]. The OA as it is found postnatally derives from the contribution of 4–5 embryonic arteries which partially regress after anastomosing together: the primitive maxillary artery (PMA), the primitive ventral ophthalmic artery (PVOA), the primitive dorsal ophthalmic artery (PDOA), the stapedial artery (StA), and, possibly, the primitive olfactory artery (POlfA) (Fig. 1).

The PMA contributes with its lateral branch to the blood supply of the optic vesicle in 4- to 5-mm embryos. It starts regressing early, in 7- to 12-mm embryo. The PMA arises from the ICA caudal to the site origin of the adult OA. In the adult, the remnant of its lateral branch may persist becoming the anastomosis occurring between the deep recurrent OA and the inferolateral trunk of the ICA [18].

The primitive ventral ophthalmic artery (PVOA) originates very high, opposite the anterior choroidal artery. It first appears in 9-mm embryos and provides the medial ciliary artery to the adult OA [51]. The PDOA forms even earlier as it is detectable in 4-mm embryos. It arises from the intracranial ICA, at the level of the posterior communicating artery and caudal to the PVOA. The PDOA supplies the lateral ciliary artery and the hyaloid artery (future central artery of the retina) to the adult OA [51]. At any rate, as recently pointed out [16], since they both arise from sites that are distal to the adult OA stemming place, the primitive OAs cannot account for the occasional intracavernous origin of the OA that, instead, should be referred to the enlargement of a persistent lateral branch of the PMA. According to Padget [51], the site of origin of the regular OA as it is found in the adult is the result of the caudal migration along the ICA of the PDOA. Indeed, the mechanism of such migration is not clear. Apparently, the stem of the adult OA is a newly formed secondary branch of the ICA that annexes the PDOA [51]. In 18-mm embryos, the PVOA and the PDOA join together within the orbit, beneath the optic nerve. By the subsequent regression of its main stem, the distal part of the PVOA (medial ciliary artery) is annexed to the OA. The PDOA, as above mentioned, migrates caudally along the ICA to reach the adult position. In the adult, the remnants of the original stems of the PDOA and of the PVOA are possibly retained as the minute branches supplying the region of the optic chiasma [51].

The POlfA is early located close to the optic vesicle. Based on studies carried out on rats, a branch of the POlfA, referred to as the recurrent POlfA, is supposed to connect the parent vessel with the PMA supplying a capillary network around the optic stalk [18]. When the development proceeds regularly, this connection is believed to last up to 12- to 14-mm stage. In the adult, this branch will eventually become the small chiasmatic rami of the anterior cerebral artery [18]. The rare infraoptic course of the anterior cerebral artery occasionally observed in the adult can be explained as the persistence of the anastomosis between the recurrent POlfA and the lateral branch of the PMA [18].

The StA makes a contribution to the orbital blood supply with its supraorbital (upper) branch that enters the orbit with its orbital end via the superior orbital fissure already in 18-mm embryos. In 16- to 18-mm embryos, the maxillomandibular (lower) division of the StA anastomoses with the maxillary artery forming the stem of the middle meningeal artery (MMA). Concomitantly, the segment of the StA medial to the stapes regresses and, thanks to the newly formed anastomosis with the maxillary artery, the StA is definitively annexed to the ECA.

## Variations of the OA origin

It is well known that the OA is the first extracavernous branch of the ICA. This is certainly true in most cases. However, some variations have been reported as the direct result of small derangements from the normal developmental program of the OA.

### OA origin from the middle meningeal artery

**Fig. 2:**
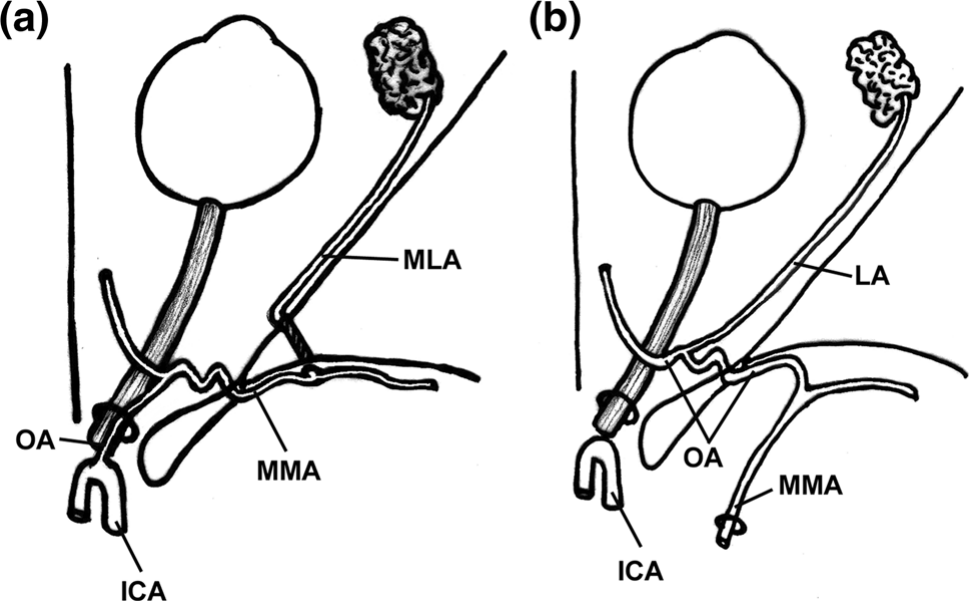
Schematic drawing of two important variations involving the anastomoses between the ophthalmic artery (OA) and the middle meningeal artery (MMA). **a** When the proximal segment of the intracranial part of the StA regresses, the MMA originates from the OA taking advantage of an anastomosis between the two vessels. **b** When its proximal segment regresses, the OA originates from the MMA taking advantage of same anastomosis mentioned in **a**. *ICA* internal carotid artery, *LA* lacrimal artery, *MLA* meningo-lacrimal artery

The intracranial part of the StA becomes in the adult the MMA. The orbital ramus of the StA is responsible for the blood supply of the extraocular structures of the orbit and enters the orbit through the superior orbital fissure. In the orbit, this artery divides into two branches, a lateral one directed to the lacrimal gland, and a medial one referred to as ethmoido-nasal artery or naso-ciliary artery [30, 45]. Variations in the development of the StA lead to several possible outcomes. When the orbital ramus fails to regress at the level of the superior orbital fissure, a connection between the MMA and the OA persists postnatally as the recurrent meningeal branch of the lacrimal artery [36, 45]. In other cases, the division of the orbital ramus occurs within the cranial cavity [45], one branch entering the orbit through the orbitomeningeal foramen (also known as Hyrtl’s foramen) and becoming the meningo-lacrimal artery, the other branch (ethmoido-nasal artery), passing through the superior orbital fissure and being annexed to the OA. If the ethmoido-nasal artery does not regress completely at the level of the superior orbital fissure, a direct anastomosis between the MMA and the OA is found in the adult and is referred to as meningo-ophthalmic artery [36, 45]. The occasional aberrant regression of the stem of the MMA or that of the proximal segment of the OA is compensated by the presence of the meningo-ophthalmic artery or by the recurrent meningeal branch of the lacrimal artery [16]: depending on the circumstances, these vessels may become an aberrant MMA originating from the OA/lacrimal artery or, more important to us, an aberrant OA arising from the MMA (OA_MMA_) (Fig. 2) [36, 45]. The stemming from the MMA is certainly the most frequently reported aberrant origin of the OA [11, 23, 39, 40, 65, 66]. In large series of dissections or radiological surveys, the OA_MMA_ has been reported in as much as 1.2–3.3% of orbits [24, 29, 64]. Sometimes, the OA_MMA_ flanks a regular OA originating from the ICA (OA_ICA_). Such occurrence is dealt with in a dedicated paragraph as it should be considered a double OA. The OA_MMA_ most frequently enters the orbit through the superior orbital fissure [36, 45]. However, this pathway may not be the only one possible. The orbitomeningeal foramen, known to transmit the meningo-lacrimal artery, can also house the recurrent meningeal branch of the lacrimal artery and, possibly, even the meningo-ophthalmic artery [13, 25, 42]. As these vessels can replace the OA, the foramen can also assume a very large caliber representing a major hazard in the surgery of the lateral wall of the orbit [42].

### Origin from the intracavernous ICA

**Fig. 3:**
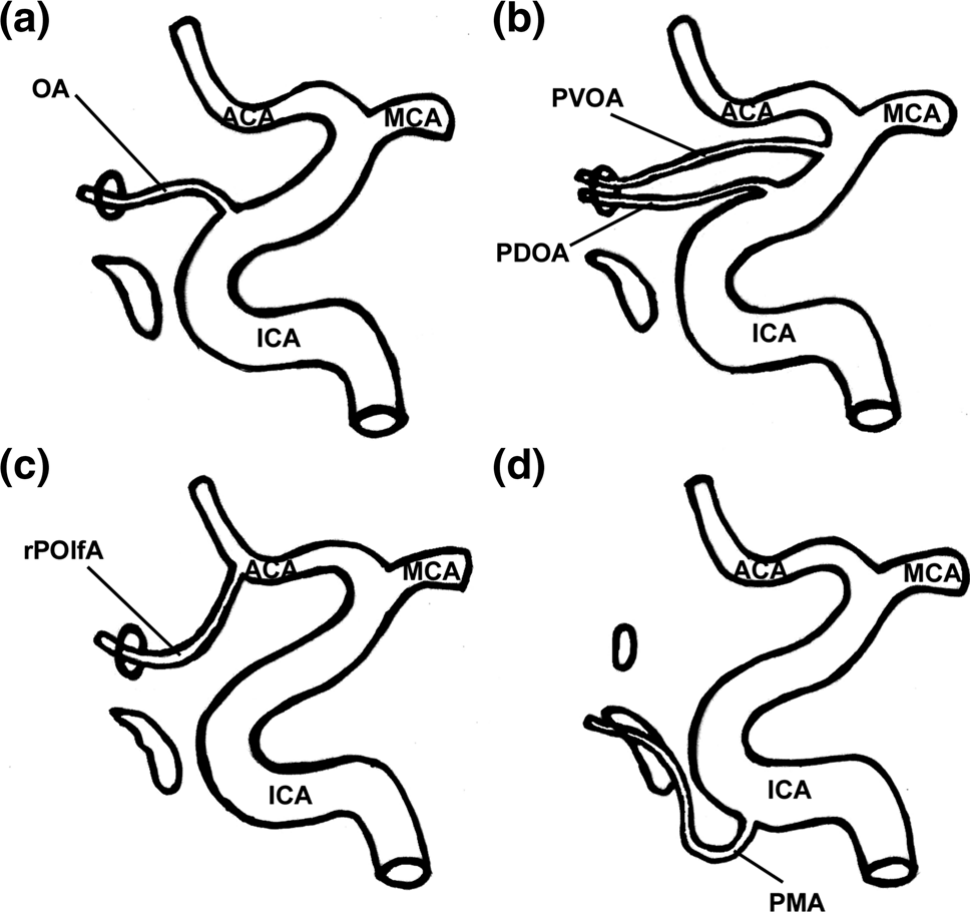
Main variations of the origin of the ophthalmic artery (OA) from the internal carotid artery (ICA). **a** OA arises from the ICA as soon as the ICA emerges from the cavernous sinus. This is the regular origin of the OA; **b** OA arises from the supraclinoid segment of the ICA. The persistence of the primitive dorsal ophthalmic artery (PDOA) or of the primitive ventral ophthalmic artery (PVOA) is likely responsible for this variation. In the adult, it is not possible to make a distinction between these two vessels; **c** OA arises from the anterior cerebral artery (ACA). This origin is likely due to the persistence of the recurrent primitive olfactory artery (rPOlfA); **d** OA arises from the intracavernous segment of the ICA. This origin is believed to be due to the persistence and enlargement of the lateral branch of the primitive maxillary artery (PMA). *MCA* middle meningeal artery

The OA can arise from the intracavernous ICA (Fig. 3). In this case, the OA enters the orbit through the superior orbital fissure or a duplication of the optic canal [25, 56]. The origin of the OA from the intracavernous ICA is usually referred to as the persistence of the PDOA [8, 63]. However, it has recently been observed that this interpretation was based on a misreading of Padget’s work [16]. Actually, according to Padget’s description, the PDOA does not originate caudally to the adult OA (i.e. from the intracavernous segment of the ICA). In embryos, the only artery arising from the cavernous ICA and supplying the eye is the PMA. In the adult, such vessel is sometimes preserved as the anastomosis occurring between the deep recurrent ophthalmic artery and the anteromedial branch of the inferolateral trunk [18, 67]. In few cases, this anastomosis is a large vessel, a condition that should be referred to as the persistence of the lateral branch of the PMA and that, instead, is wrongly known as the persistence of the PDOA [18].

An intracavernous origin of the OA (as to say a persistent lateral branch of the PMA) has been reported several times [16, 18, 24, 34, 54]. When the frequency of this variant was ascertained, it ranged between 3.3 and 8% of cases [25, 29, 56]. However, large surveys conducted on more than 1600 OAs by angiography or MR angiography suggest a much lower prevalence that ranges between 0.42 and 1.76% of orbits [27, 64]. The difference may be due to a poor sensitivity of angiography to detect this variant. Indeed, the VI of this vessel calculated on the basis of the investigations by Indo et al. [27] and Renn and Rhoton [56] is low (VI = 0.45). The VI calculated using the frequency of visualization obtained by MR angiography [64] is even lower (0.05), suggesting that the latter technique is even less sensitive to demonstrate this anomalous origin of the OA. The entrance into the orbit of an OA arising from the intracavernous segment of the ICA has been estimated as occurring through the superior orbital fissure in 5% of cases [23]. In contrast, the passage through a duplication of the optic canal has been reported in 2–3% of orbits [23, 56]. This course, however, is possibly overestimated. An investigation carried out in almost 1000 skulls found that a duplication of the optic canal occurs only in 0.65% of orbits [6]. When entering through the superior orbital fissure, the OA passes through the posterior part of the fissure, within the tendinous annulus of Zinn and medial to the oculomotor nerve [24]. Then, running between the lateral rectus and the optic nerve, the persistent PMA courses forward and about 1 cm behind the eyeball, it turns medially to cross over the optic nerve [8, 54]. This course roughly coincides with that of the deep recurrent ophthalmic artery [35].

### Origin from the supraclinoid ICA

In embryos, the PVOA and the PDOA take origin close to the terminal division of the ICA. When developing regularly, the stem of the PVOA regresses, while the stem of the PDOA migrates caudally, so that the origin and the intracranial course of the OA are established as they are normally found in adults. Sometimes, however, the caudal migration of the PDOA fails or the PVOA persists. In both cases, the persistence of one primitive OA in the adult results in the OA arising from the supraclinoid ICA with no way to make a certain distinction between the two primitive vessels (Fig. 3). At any rate, this is a very rare event that has been reported only occasionally [18, 19, 46, 52].

### Origin from the anterior cerebral artery

The aberrant stemming from the anterior cerebral artery is another rare variant of the OA origin [3, 4, 18, 20, 21, 26, 28, 37, 63]. This variation has been frequently explained with the persistence of the PVOA [3, 4, 26, 28, 37, 63], though it more probably involves a persistent recurrent POlfA (Fig. 3) [18].

### Origin from other intracranial arteries

Exceptionally, the OA has been reported to stem from other intracranial arteries like the middle cerebral artery [41], the posterior communicating artery [15, 47], the basilar artery [57–59], and the contralateral ICA [50].

### Double OAs

A double origin of the OA can be the result of a regular OA co-existing with an OA_MMA_ or with a persistent lateral branch of the PMA. The former case seems to be the commonest as in dissection-based studies, it has been reported in 2.4–3.3% of cases [23, 29]. However, a recent vast survey carried out on MR angiographies demonstrated an OA_MMA_ co-existing with an OA_ICA_ in only 0.18% of cases [64]. Once again, MR angiography seems to be scarcely sensitive to detect variations of the OA origin. The VI calculated using the values determined by Uchino et al. [64] and Hayreh [23] is in fact extremely low (0.075). Interestingly, the larger OA has been consistently reported to be the OA_MMA_ [25, 29, 44]. The two OAs usually join together, the meeting site being located either medial or lateral to the optic nerve [24]. In other instances, however, the eyeball is served by the OA_ICA,_ whereas the rest of the orbit is supplied by the OA_MMA_, the two systems apparently not having any connection [44, 45].

Two OAs arising from the ICA are really rare. To our knowledge, 11 cases have been reported so far [1, 8, 16, 18, 30, 34, 48, 49, 64, 67], though we believe that the two deep recurrent ophthalmic arteries reported by Lasjaunias [35] should be included in the count. It is frequently said that the persistent lateral branch of the PMA (previously referred to as persistent PDOA) enters the orbit through the superior orbital fissure [1, 8, 30, 48, 54, 62]. However, in some cases, the diagnosis has been merely angiographic [30, 34, 62, 64] and the course of the artery should be considered presumptive. Indeed, the persistent PMA can enter the orbit also through a duplication of the optic canal [23, 56]. In most cases, the two OAs are independent vessels with their own territory of distribution [1, 48, 64]. However, a small anastomosis has been observed between the two vessels in one case [30], whereas in few other cases, the two OAs joined together to form, distally to their anastomosis, a “common OA” [8, 16, 35]. In the latter instance, the anatomic variant should be better described as a double ICA origin of the OA. Interestingly, when two OAs co-exist together, one can lie angiographically occult [8]. This observation legitimately raises the issue on the actual frequency of this anatomic variant which, though rare, could be less exceptional than previously thought.

In one case, to the best of our knowledge, the only one so far reported, a double OA has been the result of the simultaneous presence of a persistent recurrent branch of the POlfA coming from the anterior cerebral artery and a larger OA_MMA_. Unfortunately, no information is available on the intraorbital distribution of the two vessels [4].

### Clinical considerations

On the basis of 1643 selective angiographies, an anomalous site of origin of the OA from the ICA has been associated with a 50-fold higher risk of ICA anterior wall aneurysms [27]. Ligation or embolization of the MMA or that of the maxillary artery is a procedure that can endanger the eye if the OA stems from the MMA [22, 61]. The same hazard as well as a high risk of severe bleeding can be expected in surgical procedures involving the lateral wall of the orbit when an OA arising from the MMA or a large meningo-ophthalmic artery runs through the orbitomeningeal foramen [42].

## Additional sources of blood supply to the orbit from the ECA

The blood supply of the orbit receives a contribution from several branches of the ECA which anastomose with variable frequencies with the OA [10, 23, 45]. A few branches of the ECA can also supply part of the orbit without making meaningful anastomoses with the OA. Basically, all extraocular branches of the OA can have connections with rami of the ECA. In particular circumstances, some of them represent major alternative pathways for the blood supply to the orbit and may acquire clinical relevance [42, 45]. The anastomoses between the OA and the ECA are numerous. Some of them are quite common, others are rare, and others can be considered exceptional. Unfortunately, cadaver-based studies addressing the frequency of OA-ECA anastomoses are few [7].

One recent report has shed some light on the matter, though the investigation carried out by angiography on children affected by intraocular retinoblastoma should not be considered necessarily representative of the adult orbit circulation [10]. Overall, at least one anastomosis (but many children showed more than one) can be demonstrated by angiography in 44.33% of orbits, the frequency of visualization (angiographic incidence) being dependent, however, on the technique employed. When angiography is carried out either through the OA or through the ECA, the angiographic incidence increases up to 91% of orbits suggesting that, at least in children, one connection between the OA and the ECA can be found almost always if properly searched [10].

The list of anastomoses between the OA and the ECA is long (Table 1). In general, they can be divided into anastomoses located in the posterior or in the anterior orbit [10]. A third minor group is represented by anastomoses connecting the ethmoidal arteries with arteries of the nasal cavity.

**Table 1:** Main anastomoses between the ECA and the OA

Name	OA branch	ECA branch	Anat. F.	Ang. F.	VI
	Lacrimal a.	MMA	47.14% [12]	37.7% [10]	0.80
Meningo-ophthalmic a.	OA itself	MMA	ND	31.1% [10]	–
	Lateral muscular a.	MMA	5.71% [12]	ND	–
A. of the superior orbital fissure	Deep recurrent OA	Maxillary a.	ND	3% [32]	–
	Lacrimal a.	Anterior deep temporal a.	ND	33.3% [10]	–
	Lacrimal a.	Transverse facial a.	ND	ND	–
	Lacrimal a.	Orbital branch of the infraorbital a.	ND	ND	–
	Lacrimal a.	Zygomaticoorbital a.	ND	2.22% [10]	–
	Dorsal nasal a.	Facial a.	60% [7]	8.9% [10]	0.15
	Dorsal nasal a.	Orbital branch of the infraorbital a.	27% [7]	6.6% [10]	0.24
	Supraorbital a.	Superficial temporal a.	33% [7]	2.22% [10]	0.07
	Supraorbital a.	Zygomaticoorbital a.	ND	2.22% [10]	–
	Supratrochlear a.	Superficial temporal a.	ND	2.22% [10]	–
	Anterior ethmoidal a.	Sphenopalatine a.	ND	ND	–
	Posterior ethmoidal a.	Sphenopalatine a.	ND	ND	–

*Anat. F.* anatomic frequency, *Ang. F.* angiographic frequency, *ND* not determined

### Anastomoses of the posterior orbit (always involving the MMA)

1. The anastomosis most frequently visualized by angiography (up to 37.77% of orbits) is the connection between the MMA and the lacrimal artery via its recurrent meningeal branch [10, 23, 55]. Its VI is 0.80 (Table 1) as to say that angiography demonstrates this anastomosis roughly in 4 cases out of 5.
2. A second anastomosis frequently observed on angiograms is the meningo-ophthalmic artery [10, 45]. In spite of its recurrent angiographic visualization (Table 1) and though the meningo-ophthalmic artery can be exploited for drug delivery of intra-arterial chemotherapy [5], to our knowledge, no dissection-based study has ever investigated its frequency.
3. Less frequently (5.71% of orbits), the OA is connected with the MMA via the lateral muscular artery [12], and exceptionally via the supraorbital artery or the superior muscular artery [10, 12].
4. A novel anastomosis has recently been described between the ECA and the OA. Referred to as “artery of the superior orbital fissure”, this very small branch of the maxillary artery ascends from the pterygopalatine fossa to join the anteromedial branch of the inferolateral trunk [32]. It has been detected by 3D rotational angiography in 31.25% of orbits. This is a very small artery that becomes detectable concomitantly with hypervascular parasellar lesions [32]. Its frequency in regular hemodynamic conditions is unknown. In our opinion, the name “artery of the superior orbital fissure” conveys the wrong idea that the artery enters the orbit through the superior orbital fissure. Actually, it is the anteromedial branch of the inferolateral trunk that enters the orbit to anastomose with the deep recurrent ophthalmic artery [35]. However, in 3% of orbits, the artery of the superior orbital fissure is connected with the deep recurrent OA [32] realizing an actual anastomosis between the ECA and the OA through the superior orbital fissure.

The OA-ECA anastomoses located in the posterior orbit are usually reported to pass through the superior orbital fissure [36, 45]. However, this course is not constant [16]. For instance, the recurrent meningeal branch connecting the lacrimal artery with the MMA leaves the orbit through orbitomeningeal foramen in 10.53% of orbits [13]. The demonstration that the orbitomeningeal foramen can be double, triple, or even quadruple raises the possibility that in some cases, more branches of the MMA, than just the meningo-lacrimal artery or the anastomosis with the lacrimal artery, supply the orbit through the orbitomeningeal foramen [42]. In addition, orbitomeningeal foramina can be larger than 1 mm in 12% of cases suggesting the passage of arteries distributing to large portions of the orbit [42].

### Anastomoses in the anterior orbit

1. In the anterior orbit, the branch of the OA most frequently anastomosed with the ECA is certainly the lacrimal artery. The lacrimal artery is central to the system of anastomoses connecting the OA with the ECA either in terms of frequency or variety of connections. In addition to the above-recalled anastomosis with the MMA in the posterior orbit, the lacrimal artery can be connected with the anterior deep temporal artery [10, 23, 45], the transverse facial artery [23], the orbital branch of the infraorbital artery [23], the zygomaticoorbital artery [10], and the meningo-lacrimal artery [12] (Table 1). In children, the connection with the anterior deep temporal artery is the anastomosis most often visualized by angiography [10] (Table 1). In spite of its frequent angiographic visualization (33.3% of cases) and though this pathway can be exploited for drug delivery in intra-arterial chemotherapy [5], to the best of our knowledge, no dissection-based study has ever addressed its actual anatomic incidence. The anastomosis between the lacrimal artery and the zygomaticoorbital artery has been detected by angiography in up to 2.22% of cases [10]. Connections between the lacrimal artery and the meningo-lacrimal artery are achieved through small intraglandular rami [12]. Such intraglandular anastomoses are possible only with the concomitant presence of two lacrimal arteries and their importance is negligible [12]. The anastomoses between the lacrimal artery and the transverse facial artery or the orbital branch of the infraorbital artery are rare [25].
2. The dorsal nasal artery frequently anastomoses with the facial artery via the angular artery [7, 10, 12, 23] or with the infraorbital artery [7, 10, 23]. These connections have been detected in few cases by angiography, though they can be observed with higher frequencies by dissection. Accordingly, they have very low VIs (Table 1).
3. While leaving the orbit, the supraorbital artery can make connections with the frontal branch of the superficial temporal artery [7, 10, 23], or with the zygomaticoorbital artery [10] (Table 1). In adults, the anastomosis with the frontal branch of the superficial temporal artery has been demonstrated in 1/3 of cases [7], whereas in children, the frequency of its angiographic visualization is low [10] (Table 1). The connection with the zygomaticoorbital artery has been demonstrated only by angiography [10].
4. The supratrochlear artery anastomoses with the frontal branch of the superficial temporal artery [10, 23]. This connection, detected in up to 2.22% of angiographies, can be considered a variation of the anastomosis described in 3, though it courses more medially.
5. Anastomoses involving the palpebral arteries or the muscular branches of the OA are rare and/or of negligible clinical importance [12, 36].

The anastomoses located at the anterior orbit share very low VIs (Table 1). Such values can be explained in two ways: angiography is not an efficient technique for their demonstration or they mostly develop with age. In the latter case, a reliable VI could be produced only by the use of thorough anatomo-angiographic studies carried out on adults.

### Anastomoses of the medial wall of the orbit

1. Anastomoses between the OA and the ECA may involve the ethmoidal arteries. Both the posterior and anterior ethmoidal arteries pierce the medial wall of the orbit, and are known to make small anastomoses with branches of the sphenopalatine artery, though there is no available data on their frequency [17, 23].

### Clinical considerations

Depending on the clinical context, the multiple anastomoses between the OA and the ECA may represent a useful resource to be exploited or a hazard. They can be useful when trying to find an alternative pathway to deliver intra-arterially drugs to the eye and the OA cannot be catheterized or an adverse hemodynamic hinders the anterograde flow of the blood [5]. A number of anastomoses joining the OA with the ECA also represent an obvious advantage when, establishing collateral pathways after acute occlusion of the OA, they preserve the sight in almost 85% of cases [2]. On the other hand, the same pathways may represent a risk of unwelcome passage of embolic material into the OA (and the central retinal artery) and/or, from there, into the internal carotid territory during embolization procedures of ECA branches [10, 17].

## Orbital hemodynamic: lessons from the children

**Fig. 4:**
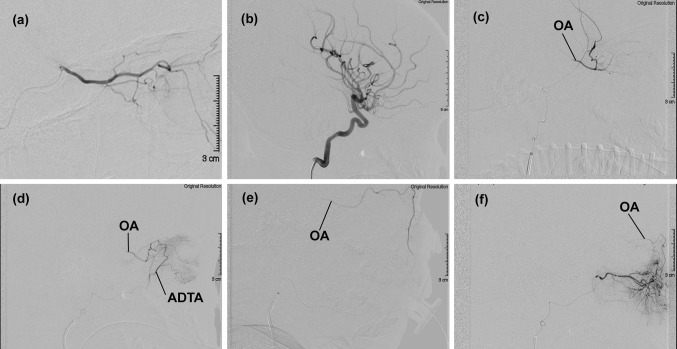
Two cases of arterial dominance. Angiographic examinations were carried out in children affected by intraocular retinoblastoma and treated with intra-arterial chemotherapy. For more details, see [5]. **a** ICA dominance. Superselective angiography of the OA highlights all major intraorbital vessels; ECA dominance. **b** Selective angiography of the ICA failed to show the OA in all sessions (*n* = 6) of intra-arterial chemotherapy on the same patient; **c** superselective angiography of the frontal branch of the MMA. The contrast medium reaches the OA via the recurrent meningeal branch of the lacrimal artery; **d** superselective angiography of the anterior deep temporal artery (ADTA). The contrast medium flows into the lacrimal artery and, from there, backward into the proximal portion of the OA; **e** superselective angiography of the facial artery. The contrast medium flows through the angular artery backward into the OA up to its origin; **f** superselective angiography of the infraorbital artery. The contrast medium flows backward into the OA through the angular artery

**Fig. 5:**
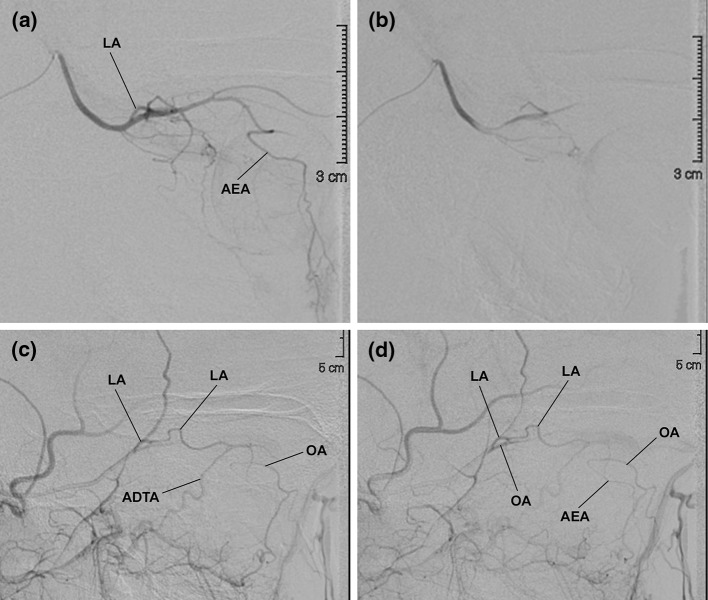
Balanced hemodynamic. Two angiographic studies carried out on the same patient demonstrate that the territory of the lacrimal artery is supplied by the ECA via the anterior deep temporal artery (ADTA). In addition, the extension of the territories of the orbit served by the OA and the ECA change between the two examinations. **a** First angiography. Superselective angiography of the OA. The contrast medium flows in almost the entire vascular tree of the OA, including the anterior ethmoidal artery (AEA). However, only a short portion of the lacrimal artery (LA) can be seen; second angiography. The hemodynamic balance between ECA and ICA is changed; **b** superselective angiography of the OA. The contrast medium does not diffuse into the OA as far as in **b** and the LA is not visible; **c**, **d** selective angiography of the ECA. The contrast medium reaches the LA via the ADTA. In the LA, the flow is forward-directed to the lacrimal gland and backward directed to the OA. In this examination, the ECA also contributes to the blood flowing into the distal OA and even into the AEA

It is always assumed that in the absence of vascular disease, the blood within the OA flows from the orbital apex towards the anterior orbital opening. As a corollary, the flow should be directed from proximal to distal also in the ramifications of the OA. However, this is not always the case. A recent study carried out on children affected by intraocular retinoblastoma who underwent several sessions of intra-arterial chemotherapy unveiled some unexpected findings [5]. In all patients, the OA was constantly present though not always visible by selective angiography of the ICA. The blood flow within the OA, in fact, was not always directed anterogradely. In greater detail, it has been observed that orbits can be entirely supplied either by the ICA, with the blood within the OA regularly flowing anterogradely (ICA dominance) (Fig. 4a), or by the ECA (ECA dominance), with the flow within the OA backward directed towards the ICA (Fig. 4b–f) [5, 43]. Between these two extreme situations (ICA or ECA dominance), a variety of possible hemodynamically intermediate conditions (balanced hemodynamic) can be found [4]. In such cases, part of the orbit is supplied by branches of the ECA and part from the ICA via the OA (Fig. 5). These three possibilities should not be considered necessarily as stable conditions [5]. When a series of angiographies is carried out monthly on the same child, it is not unusual to find different hemodynamic outlines (Fig. 5). The extension of the territories supplied by the ECA and ICA, therefore, may change in a very short time. The reason for this is unclear and it may depend just on the physiologic fine tuning of the orbit circulation. At any rate, these findings unveil that in children, a subtle balance exists between ECA and ICA, the two vessels competing for the orbital blood supply. The implications of such findings are evident particularly to the neurovascular interventionalists who desire to take advantage of the vascular flow to deliver drugs to an intraorbital target. On the other hand, on the ground of these results, one should also infer that a reversed blood flow assessed by Doppler ultrasonography within the OA of children does not necessarily mean a pathologically altered hemodynamic.

A similar in-depth investigation has never been performed by angiography on adult orbits. Indeed, most of the current knowledge on orbital hemodynamic in adults come from Doppler ultrasonography (US) studies [38]. According to such studies, in the absence of vascular pathology, an ICA dominance in the orbit seems the rule. However, though Doppler US provides valuable information [38], it does not supply a comprehensive view of the orbital circulation and some important details may escape. In particular, the ultimate source of the blood flowing within the orbit is difficult to estimate. Indeed, the direction of the flow within the OA as an indication to predict the source of the blood can be misguiding. For instance, the US assessment of blood flowing anterogradely in the distal OA does not imply that it comes from the ICA. As a matter of fact, when the OA arises directly from the MMA, the entire blood supply of the orbit ultimately comes from the ECA [25, 40]. On the other hand, even a large anastomosis between the MMA and the OA can have the same hemodynamic effects, with blood within the OA flowing anterogradely though deriving from the ECA [5]. Actually, in such cases, a reversed course within the OA might still occur in that part of the artery which is proximal to the anastomosis, a deep portion difficult to probe by US. At any rate, based also on the observations carried out on children [5, 43], not necessarily affected by retinoblastoma [43], the finding that the orbit is entirely supplied by the ECA should not necessarily imply an underlying vascular pathology, though experience says that this is highly probable.

Evidences in children show that the orbital hemodynamic can vary over the time. The balance between ECA and OA can shift in favor of one artery rather than the other in a matter of days [5]. In some children, it is possible to observe that OA-ECA anastomoses are not constantly visible. Apparently, they can functionally close or open depending on the hemodynamic requirements of the moment [5]. If their opening/closure is the cause or the consequence of the hemodynamic shift between OA and ECA dominances (including all the intermediate gradations of the balanced hemodynamic outlines) at present is impossible to say. However, results in children show that anastomoses are vascular channels that do not necessarily develop secondarily to chronic vascular disorders. In the adult, we do not have the same compelling evidences. Indeed, as we lack investigations comparable to those carried out on children (in terms of number of patients undergoing repeated angiographic studies within a relative short range of time), hemodynamic shifts between OA and ECA dominances cannot be documented at present. Nevertheless, even in adults, vascular channels may lie angiographically hidden becoming conspicuous only under particular hemodynamic circumstances [5, 16] and the presence of functionally operational anastomoses in acute settings has been demonstrated in several occasions. In particular, the absence of visual deterioration in most cases during balloon test occlusion of the ICA and/or OA before endovascular treatment of carotid-ophthalmic aneurysms confirms their ability to guarantee the blood flow to the eye even after acute OA occlusion [14, 31, 53, 60]. Hemodynamic shifts between OA and ECA dominances are, therefore, potentially feasible also in adults.

## Conclusions

If the clinician is not aware of the possible variations occurring to the site of origin of the OA, some endovascular or surgical interventions may put at risk the eye of the patient. Anomalous origin of the OA is also associated with a high risk of ICA anterior wall aneurysms.

The anastomoses that may occur between the OA and the ECA represent an anatomic resource or a hazard. They may provide alternative routes for the blood supply of the orbit but also for the unwelcome passage of embolic material during embolization procedures of ECA branches. For this reason, a detailed knowledge of their anatomy and frequency in the adults is required for the proper evaluation of the risks of some endovascular procedures. Anastomoses between the OA and ECA also influence orbital hemodynamic which in children displays a certain degree of variability, including ECA dominance of the orbital blood supply. A large-scale angiographic study of the orbital blood supply should be carried out to verify if the concept of ICA and ECA dominances as well as that of balanced hemodynamic can be extended to grown-ups as well. Finally, a comparison between children and adults is mandatory to ascertain if arterial dominances (ECA and ICA) and balanced hemodynamic are patterns that change with growth and if adult hemodynamic has the same potentials for the changes observed in children. A large-scale angiographic study carried out on adults with the same modalities previously employed on children [5] would serve the purpose. However, clinical scenarios requiring such vast investigation in adults are exceedingly rare. In spite of this, the possibility that OA/ECA anastomoses are in place, though not always visible, should be kept in mind.
